# Ultra-sensitive detection of brain-derived neurotrophic factor (BDNF) in the brain of freely moving mice using an interdigitated microelectrode (IME) biosensor

**DOI:** 10.1038/srep33694

**Published:** 2016-09-19

**Authors:** Yong Kyoung Yoo, Jaekwang Lee, Jinsik Kim, Gangeun Kim, Sunpil Kim, Jeongyeon Kim, Heejung Chun, Jeong Hoon Lee, C. Justin Lee, Kyo Seon Hwang

**Affiliations:** 1 Center for BioMicrosystems, Korea Institute of Science and Technology (KIST), Seoul 02792, Korea; 2 Department of Electrical Engineering, Kwangwoon University, 447-1 Wolgye, Nowon, Seoul 01897, Korea; 3 Center for Neuroscience and Functional Connectomics, Korea Institute of Science and Technology (KIST), Seoul 02792, Korea

## Abstract

Brain-derived neurotrophic factor (BDNF) plays a critical role in cognitive processes including learning and memory. However, it has been difficult to detect BDNF in the brains of behaving animals because of its extremely low concentration, i.e., at the sub-nanogram/mL level. Here, we developed an interdigitated microelectrode (IME) biosensor coated with an anti-BDNF an anti-BDNF antibody in a polydimethylsiloxane (PDMS)-based microfluidic channel chip. This sensor could detect BDNF from microliter volumes of liquid samples even at femtogram/mL concentrations with high selectivity over other growth factors. Using this biosensor, we examined whether BDNF is detectable from periodical collection of cerebrospinal fluid microdialysate, sampled every 10 min from the hippocampus of mice during the context-dependent fear-conditioning test. We found that the IME biosensor could detect a significant increase in BDNF levels after the memory task. This increase in BDNF levels was prevented by gene silencing of BDNF, indicating that the IME biosensor reliably detected BDNF *in vivo*. We propose that the IME biosensor provides a general-purpose probe for ultrasensitive detection of biomolecules with low abundance in the brains of behaving animals.

Brain-derived neurotrophic factor (BDNF) is a neurotrophic signalling molecule that is associated with neuronal growth, synapse maturation during development, synaptic plasticity, and axonal targeting^1^,^2^,^3^,^4^,^5^. BDNF has been implicated in a variety of psychiatric disorders, such as major depression, anxiety disorder, bipolar disorder, and other mental disorders that are related to exposure to stressful conditions^6^,^7^,^8^,^9^. Therefore, precise monitoring of BDNF in cerebrospinal fluid (CSF) has the potential to uncover important clues for development of diagnostic and therapeutic strategies for psychiatric diseases as well as neurodegenerative diseases such as Alzheimer’s disease and Parkinson’s disease^6^,^7^,^8^,^10^,^11^.

Recently, the ability to make repeated *in vivo* measurements of BDNF in the brain has emerged as an important technical challenge, as alterations of BDNF in the CSF can be caused by anxiety and stressors in daily life^12^,^13^. At present, periodic assessment of BDNF *in vivo* is accomplished via microdialysis in awake, freely moving animals with a slow collection speed of several μL/min. The resultant samples contain ultra-low concentrations of secreted BDNF in a small volume. Such small-volume samples impose technical limitations on the use of conventional methods for detecting BDNF, such as enzyme-linked immunosorbent assay (ELISA) and western blotting^12^,^13^,^14^,^15^,^16^, which require a much larger sample size.

Accuracy and consistency are the most essential criteria for biochemical detection in small-volume samples. Furthermore, because BDNF prepared via microdialysis is diluted by a factor of 10 to 100 by the perfusate (artificial CSF)^17^, high sensitivity and small sample volume consumption are required for the measurement of BDNF in CSF obtained via microdialysis *in vivo*. The microdialysis perfusion rate is typically in the range of 0.2–4 μL/min, yielding relative recoveries in the range of 10–45% 17. Even with the currently available state-of-the-art analytical techniques, microdialysis has limited time resolution (≥20 min), and samples thus still do not provide real-time information regarding changes in the neurochemical environment^18^.

In the present study, we evaluated the use of an interdigitated microelectrode (IME) sensor with a microfluidic channel for measurement of BDNF in CSF samples microdialysed from awake, freely moving mice. To maximize sensitivity and reliability, we immobilized the BDNF antibody between the electrodes and compared the IME impedance using more than three IMEs. To accommodate the evaluation of small microliter volumes, we used a polydimethylsiloxane (PDMS)-based microfluidic channel chip in the IME sensor. This sensing system was thus designed to enable simultaneous microdialysis and quantification of BDNF with high sensitivity and consistency.

## Results

**Figure 1:**
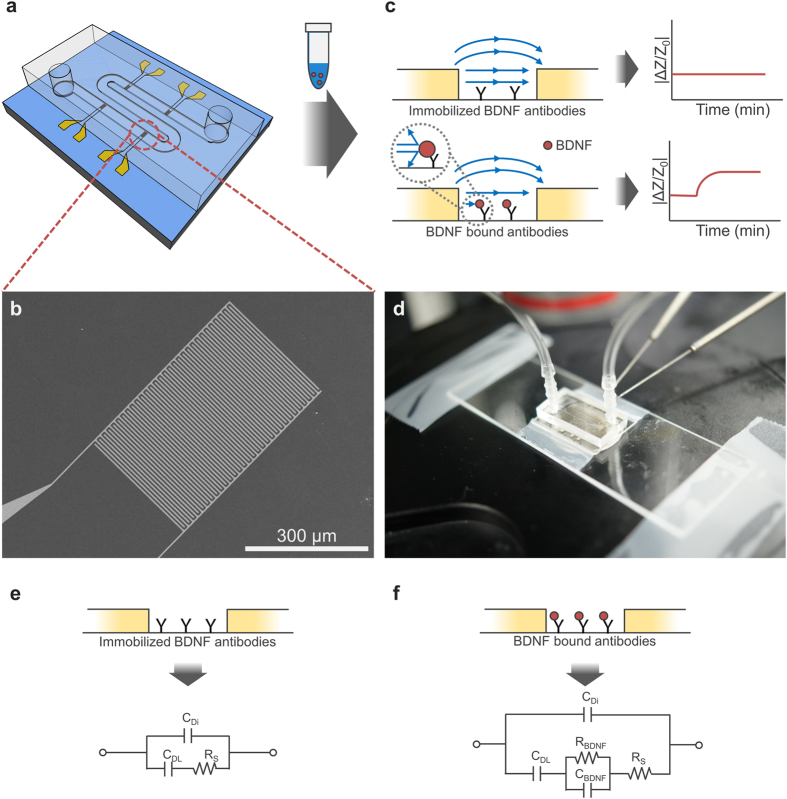
Schematic diagram for BDNF detection system using IMEs. (**a**) IMEs of 4 pairs on a chip with a PDMS microfluidic chip for the minimization of microdialysis sample volume consumption. (**b**) Scanning electron microscope image of an IME. (**c**) Illustration of impedance change elicited by the interaction of BDNF with the anti-BDNF antibody. (**d**) BDNF detection and quantification during measurement from the PDMS microfluidic channel. (**e**) Simplified equivalent circuit of the IME for BDNF detection at 100 Hz before the interaction of BDNF with the anti-BDNF antibody and (**f**) after the interaction of BDNF with the anti-BDNF antibody.

The IME sensor for BDNF detection and PDMS microfluidic channel chip for sample delivery were constructed as shown in Fig. 1a (see Experimental Procedures). The IME chip was composed of 4 IMEs. The sample volume consumption of each chip was calculated to be approximately 9 μL (2.25 μL/IME). Figure 1b shows a sample scanning electron microscope image of an IME, which consisted of a titanium/platinum electrode (30 nm/150 nm) on a silicon dioxide (SiO_2_) layer microfabricated according to the following specifications: electrode width 5 μm, gap width 5 μm, length 300 μm, with a total of 30 electrode pairs (see Experimental procedures). Figure 1c shows the sensing scheme of our IME-based BDNF detection system with immobilized antibodies for BDNF on the surface of SiO_2_ in between two neighbouring electrodes. As a first step towards the development of our BDNF sensor system, we treated the IME using 3-aminopropyl triethoxysilane, polyvinyl pyrrolidone-aldehyde, and glutaraldehyde, sequentially. Then, the IME sensor was functionalised with an anti-BDNF antibody (SC-546, Santa Cruz Biotechnology) by immobilization of the antibody on the aldehyde group of the modified substrate^14^,^19^ (see Experimental Procedures). When then samples were loaded, increases in the impedance of receptor-functionalized IMEs (impedance sensors) corresponded to the amount of the bound target molecule^20^, in this case, BDNF. The actual changes in impedance (ΔZ) were measured using an electrochemical impedance spectrometer (PGSTAT302N, Metrohm Autolab or IME analyzer, Cantis Incorporation) connected to the IME chip by two metal electrodes (Fig. 1d), and were expressed as normalized impedance changes (ΔZ/Z_0_) (Fig. 1c).

The impedance change represents the additional resistance and capacitance attributed to the BDNF molecules that are bound to the immobilized anti-BDNF antibodies (Fig. 1e,f). In the equivalent electrical circuit for the IME sensor before and after the specific interaction between BDNF and anti-BDNF antibodies on the SiO_2_ surface of the IME sensor, C_Di_, C_DL_, and C_BDNF_ are the dielectric capacitance, the electrical double layer between the buffer solution and the electrodes, and the capacitance of BDNF after the interaction, respectively. R_BDNF_ and R_S_ represent the resistance of BDNF and the resistance of the media solution, respectively. When the specific interaction between BDNF and its antibodies occur on the IME sensor surface, the impedance of IME sensor is predominantly changed by C_BDNF_ and R_BDNF_ generated by the binding of BDNF molecules to the antibodies. The impedance change thus enables prediction of the quantity of BDNF in the solution collected by microdialysis from mouse brains.

**Figure 2:**
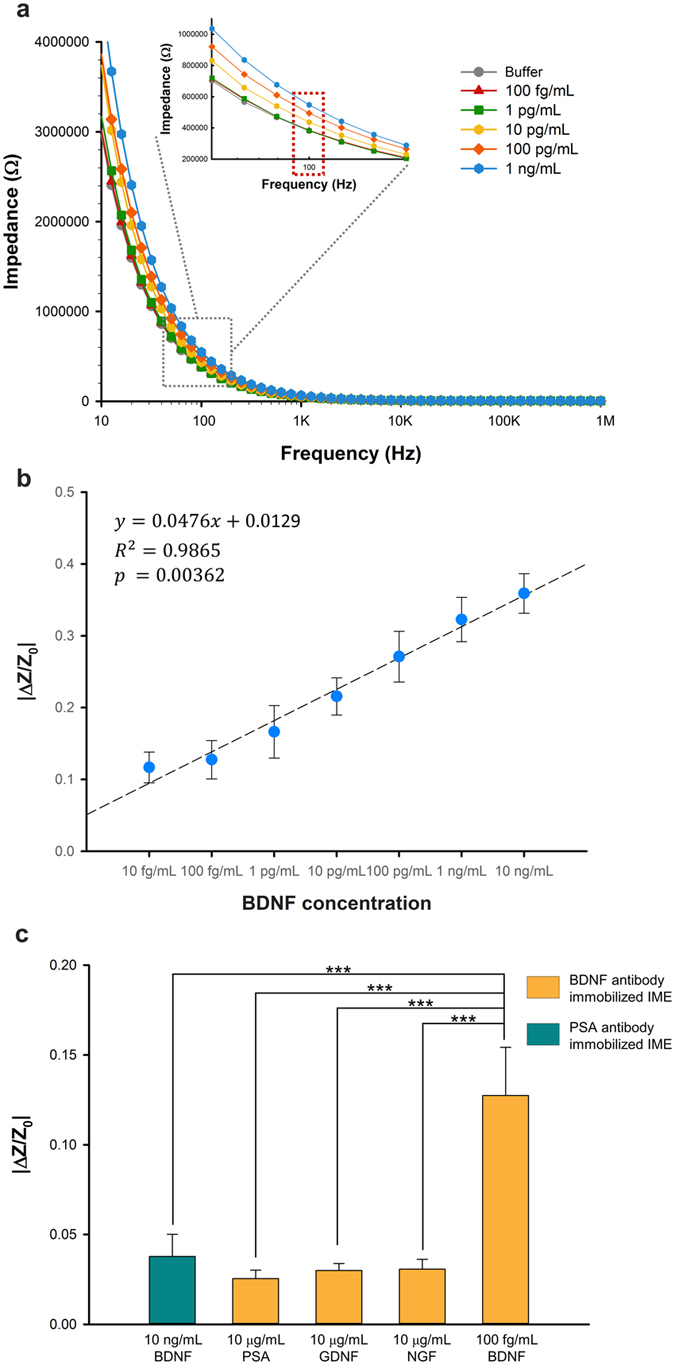
IME impedance changes elicited by biomolecular interactions. (**a**) Impedance spectra of various BDNF concentrations (100 fg/mL to 1 ng/mL) at different frequencies ranging from 10 Hz to 1 MHz. (**b**) BDNF sensitivity of the IME sensor with a range of 10 fg/mL to 10 ng/mL for microdialysis samples (n = 5). The impedance change had a linear relationship with BDNF concentration. The indicated linear equation (unit of x is in fg/mL), coefficient of variation, R^2^, and probability value (p) represent the outputs of the linear regression analysis. (**c**) Impedance changes indicating IME biomolecular selectivity for BDNF (n = 5). The impedance change of anti-BDNF antibody-immobilized IME is greater in the presence of BDNF than in the presence of other biomolecules (one-way ANOVA and *Tukey test*). (***p < 0.001, n = 5 each condition).

To evaluate the dynamic range of IME sensors for BDNF detection, solutions containing BDNF at various concentrations (10 fg/mL to 10 ng/mL) were injected into the IME chip. After BDNF was allowed to interact with BDNF antibody for 20 min, the IME was washed with buffer solution, and subsequent impedance spectra at different frequencies ranging from 10 Hz to 1 MHz were recorded after the biomolecular interaction between BDNF and its antibodies occurred on the IME sensor surface (Fig. 2a). According to the general principle^21^, impedance changes in the IME structure can occur according to the electrical and physical properties of the biomolecular interactions around the electrodes as shown in Fig. 1c. The optimum frequency can be used to efficiently detect BDNF based on the relationship between impedance and frequency. As expected, the impedance changes for different concentrations of BDNF showed different responses according to the frequency, as shown in Fig. 2a. Larger impedance changes were obtained at lower frequencies of approximately 100 Hz than at higher frequencies over 1 kHz. After considering the larger impedance changes at lower frequencies and chip-to-chip variation among IME sensors, we selected the frequency of 100 Hz for optimal BDNF detection (Fig. 2a inset).

Using this method, we evaluated the sensitivity and selectivity of the IME sensor. We observed average impedance increases of 0.116 ± 0.021, 0.127 ± 0.026, 0.166 ± 0.036, 0.215 ± 0.026, 0.271 ± 0.035, 0.322 ± 0.030, and 0.359 ± 0.027 following the injection of 10 fg/mL, 100 fg/mL, 1 pg/mL, 10 pg/mL, 100 pg/mL, 1 ng/mL, and 10 ng/mL samples, respectively (mean ± standard error of mean, each concentration n = 5, sample volume = 9 μL; Fig. 2b). These results demonstrated a linear correlation between BDNF concentration and normalized impedance change based on linear regression analysis (coefficient of correlation, R^2^ = 0.9849, p = 0.00362; Fig. 2b). R^2^ = 0.9849, i.e., 98% of the total variation in impedance change could be explained by the linear relationship between BDNF concentration and impedance change. Because of the dilution occurring during microdialysis, we expect the observed concentration of BDNF to be approximately 100-fold lower than the typical concentration of BDNF in mouse CSF, which has been reported to be in the range of pg/mL to ng/mL^13^,^15^,^22^. Therefore, the observed detection range falls well within the expected concentration range for BDNF diluted in the microdialysate.

To test the selectivity of the IME sensor, we prepared an IME functionalized with an antibody against prostate-specific antigen (PSA) and evaluated BDNF detection. We selected PSA and its antibody as a control antigen-antibody pair because PSA, which has a strong affinity for the anti-PSA antibody (K_D_ = 504 pM), is typically used for the study of antigen-antibody interactions^23^. The anti-PSA antibody is also known for its lack of cross-reactivity with other biomolecules^23^,^24^. We found an average impedance change of 0.0385 ± 0.010 (n = 5) for 10 ng/mL BDNF against anti-PSA antibody-immobilized IME (Fig. 2c, green bar). This value was significantly different from that obtained with 100 fg/mL BDNF against anti-BDNF antibody-immobilized IME (p < 0.001, one-way ANOVA). Furthermore, we measured impedance changes from an IME functionalized with BDNF following injection of PSA, glial cell line-derived neurotrophic factor (GDNF), and nerve growth factor (NGF). We found that 10 μg/mL PSA, 10 μg/mL GDNF, and 10 μg/mL NGF produced significantly lower impedance increases of 0.0286 ± 0.005 (n = 5), 0.0307 ± 0.003 (n = 5), and 0.0310 ± 0.004 (n = 5), respectively, compared to 100 fg/mL BDNF against the anti-BDNF antibody-immobilized IME (p < 0.001, one way ANOVA; Fig. 2c). Although the concentrations of the PSA and other growth factor were approximately 100 million-fold higher, the impedance increase following the injection of 100 fg/mL BDNF (0.125 ± 0.022, n = 4) was 3 times greater than that of 10 μg/mL PSA, 10 μg/mL GDNF, and 10 μg/mL NGF. These results indicate that the IME sensor platform shows ultra-sensitivity even at fg/mL concentrations of BDNF and high selectivity over other growth factors.

**Figure 3:**
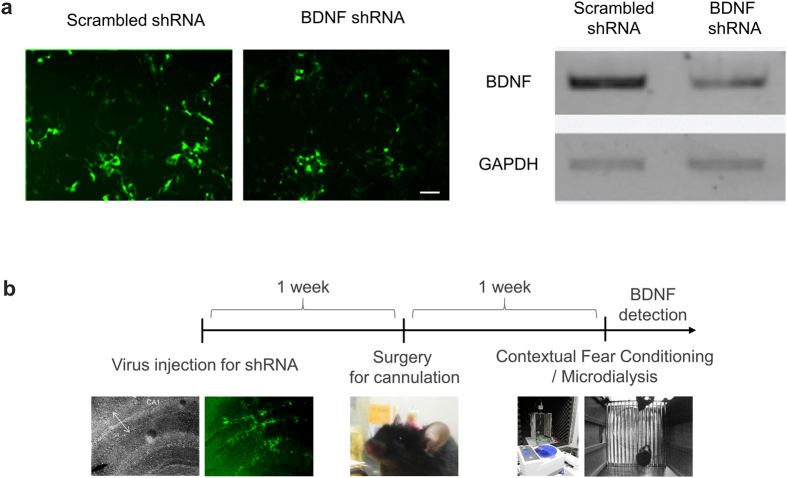
Development of anti-BDNF shRNA and *in vivo* gene silencing of BDNF using lentivirus. (**a**) Validation of BDNF shRNA. Upper panel shows exemplary images of cultured HEK293T cells transfected with SC or BDNF shRNA candidates. mCherry and GFP were used as fluorescent reporters for BDNF and shRNA expression, respectively. Scale bar (white dash) indicates 30 μm. Middle panel shows result of RT-PCR of BDNF mRNA for each shRNA candidate. Lower panel shows bar graph of knockdown efficiency for each BDNF shRNA candidate. (**b**) Experimental procedure for BDNF detection from an animal *in vivo*.

**Figure 4:**
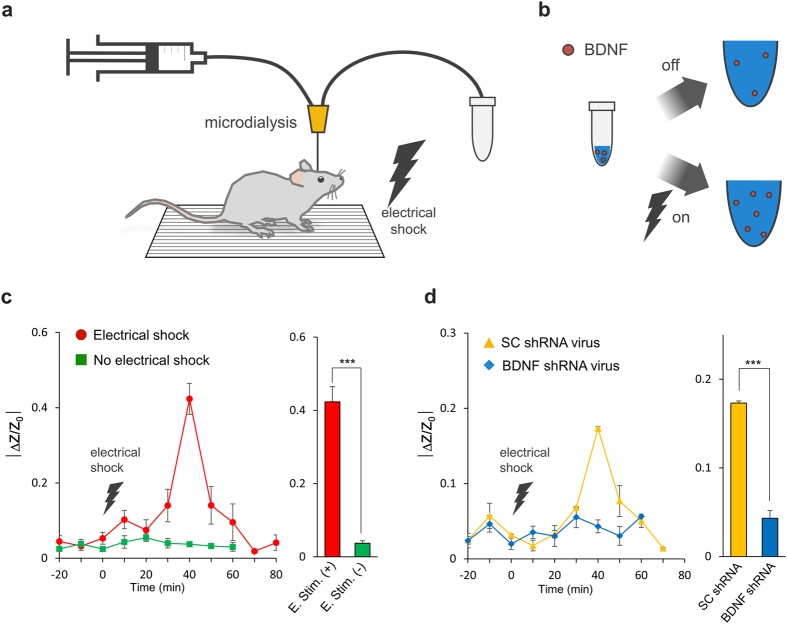
*In vivo* detection of CSF BDNF from microdialysates. (**a,b**) Schematic diagram of *in vivo* microdialysis and BDNF sample collection with or without electrical shock. (**c**) Time course of impedance changes during CSF sampling as measured by normalized impedance change from naïve mice (n = 3 per group, n = 3 IMEs per sample). Summary bar graph for changes in CSF BDNF levels 40 min after electrical shock. (**d**) Time course of impedance changes from SC and anti-BDNF shRNA-injected mice during CSF sampling. (***p < 0.001).

Finally, to assess the feasibility of using the IME sensor for *in vivo* detection of BDNF, we performed microdialysis in the brains of freely moving mice to assess the changes in BDNF over time in a learning and memory task. BDNF is known to be released in the hippocampus during intense learning and memory tasks such as the context-dependent fear conditioning test^25^. To specifically control BDNF expression *in vivo*, we first developed a short-hairpin-forming interference RNA (shRNA) specifically targeting mRNA for BDNF. This anti-BDNF shRNA knocked down BDNF mRNA with an efficacy of 73.5% compared to the control scrambled-sequence shRNA in cultured astrocytes (Fig. 3a). This BDNF-specific shRNA was cloned into a viral vector that was packaged as a lentivirus and then injected into the mouse hippocampus to locally silence the production of BDNF *in vivo* (Fig. 3b). One week after virus injection, we prepared the injected mice for microdialysis surgery as shown in Fig. 3b. To elicit changes in BDNF levels, an electrical shock was administered as an external stimulation to induce hippocampus-dependent contextual fear memory in mice. During the memory task, mouse CSF was continuously microdialysed and sampled in 20-μL volumes collected over 10 min (collection speed: 2 μL/min) (Fig. 4a), and BDNF was detected in each sample using IME sensors (Fig. 4b). We found that basal level changes in impedance were lower than 10% in all mouse groups (−40~0 min). In naïve mice (normal mice without injection of virus), administration of electrical shock elicited a dramatic change in the average impedance of up to 0.42 (0–20 min); the impedance later returned to baseline levels (Fig. 4c). However, no changes in impedance over time were observed in the control no-shock group (Fig. 4c). The peak level of BDNF was significantly higher in mice with electrical shock than in control mice (p > 0.001, unpaired t-test, Fig. 4b). Based on calibration data obtained prior to experiments, the maximum concentration of BDNF detected via impedance change was approximately 100 pg/mL in the naïve group. However, in mice injected with BDNF shRNA, electrical shock did not elicit any significant changes in impedance (Fig. 4d), whereas in mice injected with control scrambled shRNA, electrical shock elicited a similar time course of BDNF increase and decrease as in the naïve group (Fig. 4d). The peak level of BDNF was significantly reduced in the BDNF shRNA group compared to the scrambled shRNA group (p > 0.001, unpaired t-test, Fig. 4d). These results indicate that the IME sensor provides reliable detection of extracellular BDNF levels *in vivo*.

## Discussion

Despite its importance in brain function and neurological diseases, BDNF has been difficult to study because of the lack of sensitive methods to detect it at low levels. We have developed an ultra-sensitive IME sensor with a microfluidic channel for measurement of BDNF in microdialysed CSF samples from the hippocampus. To maximize sensitivity and consistency, we immobilized an anti-BDNF antibody between the electrodes and measured the changes in IME impedance. To accommodate the evaluation of small microliter volumes, we used a polydimethylsiloxane (PDMS)-based microfluidic channel chip on the IME sensor. This sensing system was designed to enable simultaneous microdialysis and quantification of BDNF with high sensitivity and selectivity, with one-step direct binding of BDNF to the anti-BDNF antibody (without need for a secondary antibody step). Using this device, we achieved pg/mL sensitivity for detection of BDNF and high selectivity over other growth factors, with a minimal sample volume of 9 μL.

This newly developed device enabled us to detect, for the first time, the changes in BDNF levels in the brains of freely moving, behaving mice with a time resolution of 10 min per sample. With this time resolution, we demonstrated for the first time that BDNF levels were dramatically and transiently increased in naïve mice with a maximal peak at 20 min post-shock, and they subsequently returned to the baseline level. This delay (20 min post-shock) in the time course of BDNF release in response to electrical stimulation is consistent with previous observations that BDNF is involved in late long-term potentiation (LTP) rather than in early LTP^26^,^27^.

The maximum concentration of BDNF in the microdialysed samples was estimated to be approximately 25 ng/mL. The baseline level was estimated to be 100 fg/mL. This ultra-low level of BNDF is virtually impossible to detect using conventional methods such as ELISA. After correcting for the dilution factor^17^ of approximately 10 to 100^28^,^29^,^30^,^31^,^32^, the *in vivo* concentration of BDNF was estimated to be 1–10 pg/mL at baseline and 250–2,500 ng/mL at maximum. These values are comparable to the previously reported value of ~85 ng/mL in a dialysate collected from the mouse hippocampus^33^. We further demonstrated that increases in BDNF elicited by electrical shock were eliminated by genetic knockdown of BDNF with BDNF shRNA but not with control SC shRNA (Fig. 4c), validating the specificity and reliability of our IME-based system for *in vivo* detection of BDNF.

In conclusion, we demonstrate the feasibility of the impedimetric BDNF sensor in the assay of microdialysis samples from awake, freely moving mice. Our system offers high specificity in tandem with small sample volume consumption and fewer steps in the detection procedure. The implementation of this system resulted in the finding that external stimulation elicits transient alterations of extracellular BDNF level in the CSF of mice. Our highly sensitive and sample-efficient system has potential for the future study of biological correlates of stress and behaviour in mice as well as human subjects, and can be expanded to evaluate a variety of biomolecules in models of neurological and neurodegenerative diseases with readily available antibodies. Moreover, the utility of this detection strategy for analytical and diagnostic systems in clinical settings should not be overlooked and should be the focus of future research efforts.

## Methods

### IME biosensor and microfluidic channel chip fabrication

For the insulating layer, a 300 nm silicon dioxide (SiO_2_) layer was grown on a silicon (Si) wafer using thermal oxidation. Platinum (Pt, 150 nm) and titanium (Ti, 30 nm) were deposited by sputtering to form an adhesion layer. The Pt/Ti electrode was patterned using conventional photolithography equipment (MA6, Karl Suss) and etched using an inductively coupled plasma-reactive ion etcher (Oxford Instruments). A structure with a width of 5 μm, length of 300 μm length, gap of 5 μm, and 30 finger pairs was formed on the SiO_2_ layer. A polydimethylsiloxane (PDMS) microfluidic channel chip (channel width 1 mm, height 50 μm, length 3 cm) was fabricated. The reservoir of the PDMS chip for sample injection was formed with a 1-mm diameter and 3-mm depth. The calculated channel volume was approximately 1.5 μL, and the calculated reservoir volume was approximately 2.3 μL. However, because of inherent sample loss during loading, we calculated an approximate sample consumption of 9 μL for BDNF detection.

### Functionalization of IME biosensor and BDNF reaction

To remove contamination and form hydroxyl groups for functionalization of the SiO_2_ surface (i.e., the recognition layer), the fabricated IME was treated with piranha cleaning (5:1 ratio of sulfuric acid (H_2_SO_4_) and hydrogen peroxide (H_2_O_2_)) for 30 min. For the formation of amine functional groups on the surface, the cleaned SiO_2_ surface was immersed in 3-(ethoxydimethylsilyl)propylamine solution (APMES; 1% in isopropyl alcohol (IPA); Sigma-Aldrich) for 3 h. The IME surface was then washed with IPA, a 100 mM NaHCO_3_ solution, and deionized water. The IME chip was dipped and stirred in polyvinyl pyrrolidone-aldehyde solution (PVP-CHO; 10 mM in 100 mM NaHCO_3_ solution; pH 9.0) for 6 h. Next, the IME chip was washed with 100 mM NaHCO_3_ and deionized water. We then immersed the IME chip in 10 mM sodium borohydride (NaBH4, in 100 mM NaHCO_3_) for 1 h. Finally, to form an antibody linker, the IME chip was dipped in a 1% glutaraldehyde solution. After the formation of aldehyde groups on the SiO_2_ surface, the chip was immersed in 10 μg/mL BDNF antibody in 1X phosphate buffered saline (SC-546, Santa Cruz biotechnology) for immobilization. For reaction of BDNF with its antibody, recombinant BDNF (eBiosicence Inc. San Diego, CA) was diluted in artificial cerebrospinal fluid (ACSF) buffer to obtain a target concentration of 100 fg/mL to 10 ng/mL. The diluted recombinant BDNF (9 μL) was injected to anti-BDNF antibody-functionalized IME in the PDMS microfluidic channel and reacted for 20 min. After the BDNF reaction, the IME was washed with ACSF buffer.

### Impedance measurement

The interaction between BDNF and the anti-BDNF antibody was measured using an impedance measurement system (PGSTAT302N, Metrohm Autolab & IME analyzer, Cantis Incorporation) for IME sensing. The probes of impedance measurement system connected on the IME electrode pattern. The AC voltage of 10 mV applied to the electrodes produced both conduction and displacement current through the sample. We swept the frequency from 10 Hz to 1 MHz. For observation of impedance change resulting from the interaction between the anti-BDNF antibody and BDNF, we measured the bode presentation of the impedance modulus before the interaction. To measure the BDNF level, samples were collected from mice using microdialysis and injected onto the IME chip. After the interaction, the IMEs chip was washed using buffer solution. The bode presentation of the IME impedance was measured. We also intensively monitored the ratio of the impedance change of BDNF-reacted IMEs.

### Microdialysis and contextual fear conditioning

Mice (B6/C3) were anesthetized with 2% avertin (20 μL/g, i.p.) and mounted on a stereotaxic frame (Stoelting). After the skull was exposed, a burr hole was drilled, and a CMA7 guide cannula (CMA Microdialysis) was positioned in the right hippocampus (AP, −1.8 mm; ML, −1.6 mm; DV, −0.8 mm from bregma) and secured to the skull with anchor screws and acrylic dental cement. Following recovery from surgery, a CMA7 microdialysis probe of concentric design (CMA Microdialysis) was inserted through the guide cannula and into the hippocampus. The probe was connected to a CMA470 microperfusion fraction collector (CMA Microdialysis) with polyethylene (PE-20) tubing, and ACSF (purchased from CMA, Sweden) composed of (in mM) 147 NaCl, 2.7 KCl, 1.2 CaCl_2_, 0.85 MgCl_2_ (pH = 7.4) was perfused at a rate of 2 μL/min. The perfusate was collected in plastic vials in a cold chamber and stored at −70 °C until analysis. Samples were collected over 10-min intervals for more than 2 h.

Mice were tested using the FreezeFrame system (Coulbourn Instruments). For training, mouse test cages equipped with stainless-steel shocking grids were connected to a precision feedback current-regulated shock system (Coulbourn Instruments). Behaviour was recorded using a low-light video camera. Stimulus presentation was automated using Actimetrics FreezeFrame software (version 2.2; Coulbourn Instruments). All equipment was thoroughly cleaned with 70% ethanol and water between test sessions. Each test session was conducted as follows: mice were habituated for 2 min on the conditional chamber. During the test period, CSF was collected over 10 min intervals for 150–170 min (collection rate 2 μL/min, 20 μL/vial). At 90 minutes, an electrical shock was delivered 3 times (0.7 mA for 1 sec, ISI of 30 sec) from a metal floor grid. All animal experiments were performed in accordance with the National Institutes of Health Guide for the Care and Use of Laboratory Animals (NIH Publications No. 8023, revised 1978) and the Institutional Animal Care and Use Committee of the Korea Institute of Science and Technology (KIST; Seoul, Korea). The animal studies were approved by the Institutional Animal Care and Use Committee of the Korea Institute of Science and Technology.

### BDNF measurement from microdialysates

We measured the BDNF level using the IME system in one day. In Fig. 4c,d, each line and scatter plot consisted of four groups of mice: electrical shock, no electrical shock, SC shRNA virus, and BDNF shRNA virus. Each line and scatter plot shows the average value of the BDNF level in the CSF of 3 mice (each group) as determined using microdialysis. The microdialysed BDNF samples were continuously collected from each mouse. Every sample from a mouse was subjected to bioassay by 3 IMEs on a chip in one day. For BDNF detection, we did not reuse IMEs chips.

### Production of lentivirus expressing BDNF shRNA

For screening of BDNF shRNA candidates, target sequences were designed using BLOCK-iT RNAi Designer (Invitrogen, https://rnaidesigner.thermofisher.com), which provided four putative candidates for BDNF shRNA. The target regions of the shRNA candidates are as follows:

BDNF shRNA1: 5′-GCGCCCATGAAAGAAGTAAAC-3′

BDNF shRNA2: 5′-GGTGATGCTCAGCAGTCAAGT-3′,

BDNF shRNA3: 5′-GGAGCCTCCTCTACTCTTTCT-3′

BDNF shRNA4: 5′-GGTCACAGTCCTAGAGAAAGT-3′.

Synthesized oligonucleotides of each candidate for BDNF shRNA were annealed and ligated into the pSicoR-GFP vector using HpaI–XhoI restriction enzyme sites (Addgene). A scrambled-sequence shRNA-containing pSicoR construct was used as control, and the constructs were verified by sequencing. To validate the shRNA candidates, we transfected each shRNA construct and a BDNF-mCherry vector into HEK293T cells as a ratio of 1:2. The fluorescence of mCherry reflected the exogenous BDNF expression in response to the shRNA candidates. Knockdown efficiency was measured by RT-PCR analysis by analysing the level of BDNF mRNA (Fig. 3). Among four candidates, BDNF shRNA2 was the most effective in silencing BDNF gene with knockdown efficiency of 73.5% compared with SC shRNA (calculated percentage after normalization against each GAPDH intensity). The lentivirus expressing BDNF shRNA2 was produced.

### Viral injection

C57BL/6 mice (7–8 weeks) were anesthetized with 2% avertin (20 μL/g, i.p.) and mounted on a stereotaxic frame. Lentiviral pSicoR SC or BDNF shRNA was loaded in a microdispenser and injected into the hippocampus at a rate of 0.2 μL/min (total volume 2 μL) using a syringe pump and a 25 μL syringe. The stereotaxic coordinates of the injection site were as follows: AP, −1.8 mm; LM, −1.6 mm; DV, −1.70 mm from bregma.

## Additional Information

**How to cite this article**: Yoo, Y. K. *et al*. Ultra-sensitive detection of brain-derived neurotrophic factor (BDNF) in the brain of freely moving mice using an interdigitated microelectrode (IME) biosensor. *Sci. Rep.* **6**, 33694; doi: 10.1038/srep33694 (2016).
